# Supplementary material for: Voxel-wise and spatial modelling of binary lesion masks: Comparison of methods with a realistic simulation framework
Source: Neuroimage. 2021 Aug 1;236:118090. doi: 10.1016/j.neuroimage.2021.118090 (PMC8752964; doi:10.1016/j.neuroimage.2021.118090)
Supplement: Supplementary Data S1 — Supplementary Raw Research Data. This is open data under the CC BY license http://creativecommons.org/licenses/by/4.0/ [file mmc1.pdf]

Supplementary material.
Voxel-wise and spatial modelling of binary lesion masks: Comparison of methods with a realistic simulation framework

Petya Kindalova, Ioannis Kosmidis, Thomas E. Nichols

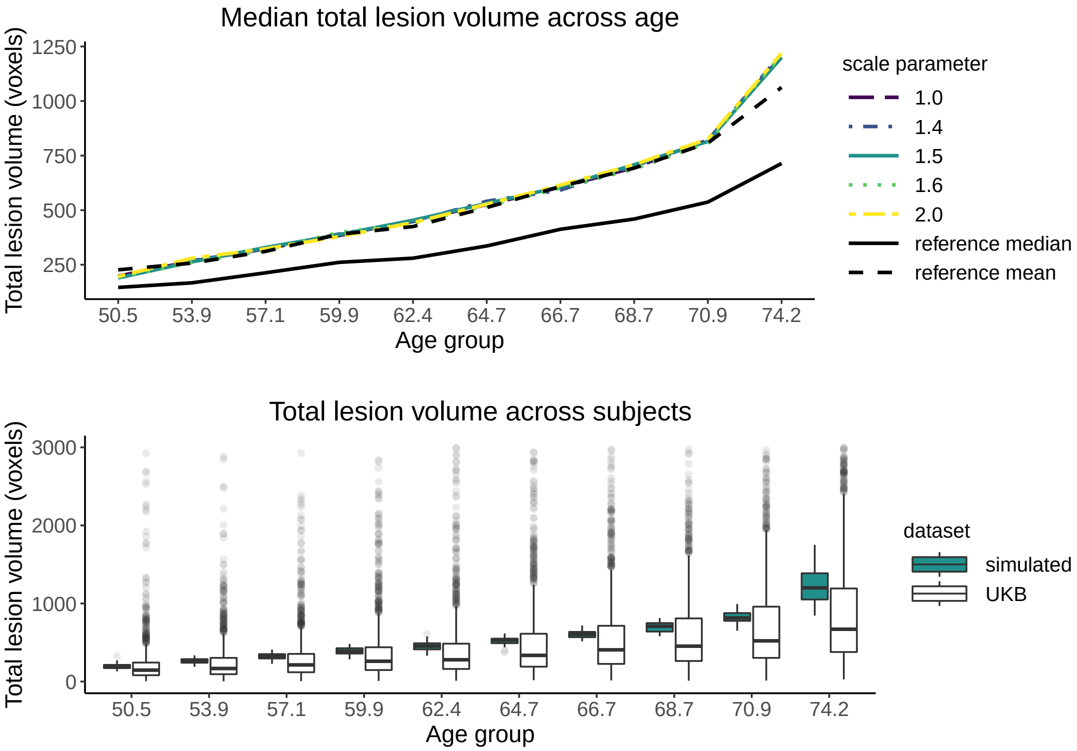

Figure S1: Gaussian random field parameter tuning by matching the reference data (UKB) median total lesion volume across age bins (black solid line). (Top) Plot of median total lesion volume across age bins for five simulation settings (five GRF scale parameter values) and reference data values (black lines). Legend values indicate the scale parameter value ℓ used to simulate a GRF for each subject in the simulated sample. (Bottom) Boxplots of total lesion volume in UKB participants (white) and in one simulated 1000-subject sample with GRF scale parameter ℓ=1.5 (blue) across ten age bins. Note the x-axis labels denote the center of each age bin, the y-axis units are in 2mm^3 voxels, and the variance GRF parameter is fixed to 1 for all simulation settings.

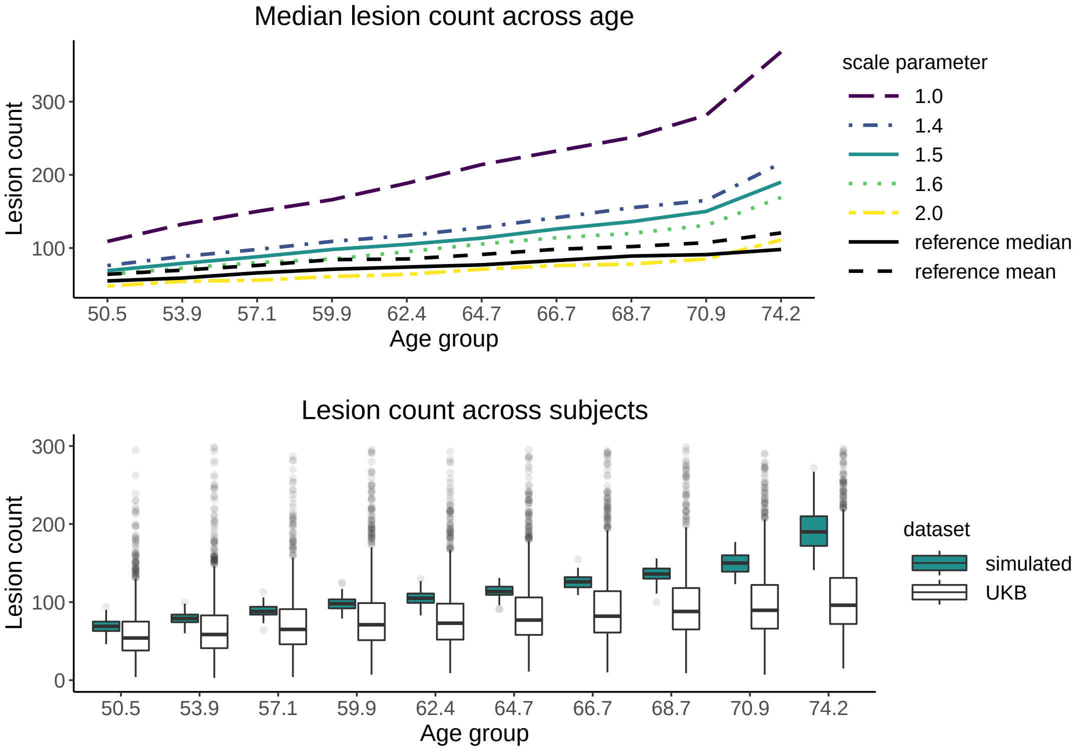

Figure S2: Gaussian random field parameter tuning by matching the reference data (UKB) median lesion count across age bins (black solid line). (Top) Plot of median lesion count across age bins for five simulation settings (five GRF scale parameter values) and reference data values (black lines). Legend values indicate the scale parameter value ℓ used to simulate a GRF for each subject in the simulated sample. (Bottom) Boxplots of lesion count in UKB participants (white) and in one simulated 1000-subject sample with GRF scale parameter ℓ=1.5 (blue) across ten age bins. Note the x-axis labels denote the center of each age bin, the y-axis units are in number of connected components (lesions), and the variance GRF parameter is fixed to 1 for all simulation settings.

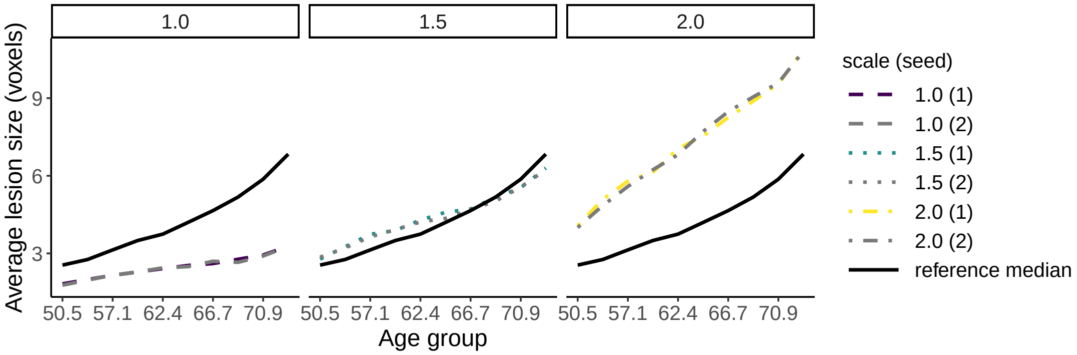

Figure S3: Gaussian random field parameter tuning by matching the reference data (UKB) median average lesion size across age bins (black solid line) replicated for two seeds. Legend values indicate the scale parameter value ℓ used to simulate a GRF for each subject in the simulated sample and the seed in brackets. The lesion summaries do not vary substantially between seeds. Note the x-axis labels denote the center of the age bins, the y-axis units are in 2mm^3 voxels, and the variance GRF parameter is fixed to 1 for all simulation settings.

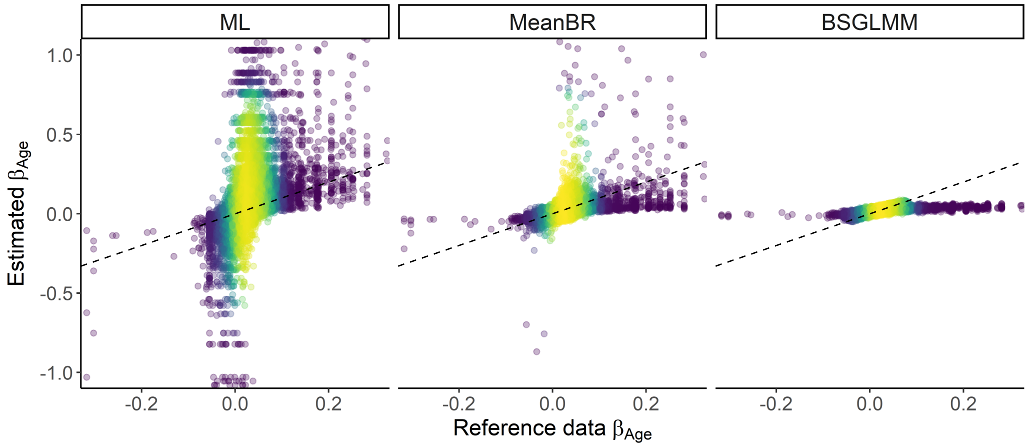

Figure S4: Estimated coefficients $\hat{\boldsymbol{\beta}}_{\text{Age}}$ (ML), $\tilde{\boldsymbol{\beta}}_{\text{Age}}$ (MeanBR), $\boldsymbol{\beta}^{*}_{\text{Age}}$ (BSGLMM) vs. $\boldsymbol{\beta}_{\text{Age}}$ (reference). Each point is coloured according to the density of points in an invisible grid overlaid on the plots (the brighter the colour, the higher the density of the points) and the identity superimposed (dashed black line). Bias reduction and the effect of the prior result in shrinkage of the coefficients towards zero with the Bayesian model following the equality line most closely. The 'horizontal effect' observed mostly at the BSGLMM plot (826 voxels have reference data coefficients greater than 0.1 in absolute value) occurs when the lesion incidence is very low. One simulated data set of 1000 subjects used; 40,338 voxels with finite MLEs plotted.

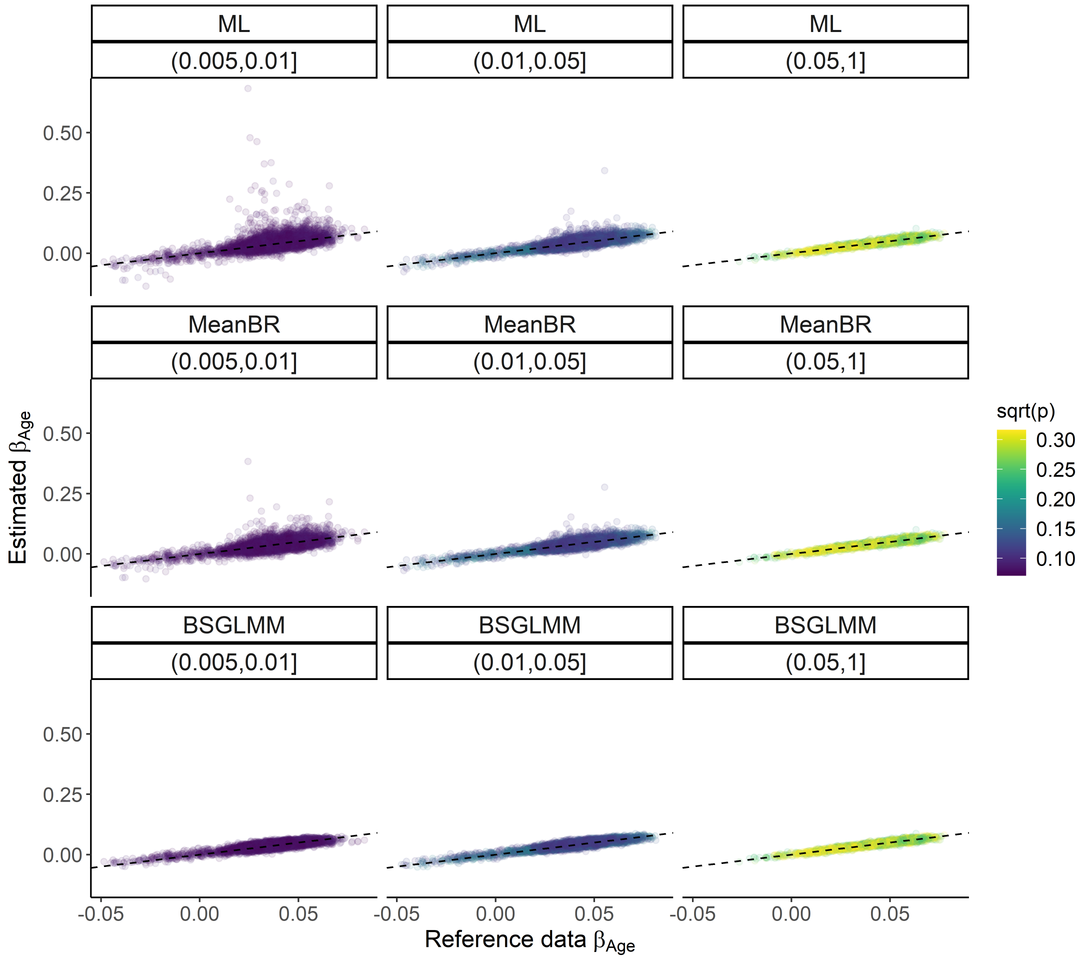

Figure S5: Estimated coefficients $\hat{\boldsymbol{\beta}}_{\text{Age}}$ (ML), $\tilde{\boldsymbol{\beta}}_{\text{Age}}$ (MeanBR), $\boldsymbol{\beta}^{*}_{\text{Age}}$ (BSGLMM) vs. $\boldsymbol{\beta}_{\text{Age}}$ (reference) across bins of voxels. Each point is coloured according to the square-root lesion probability $\sqrt{p}$ suggesting shrinkage is observed for voxels with low lesion incidence. One simulated data set of 1000 subjects used; 11,632 voxels with reference data lesion incidence $p > 0.005$ and finite MLEs plotted.

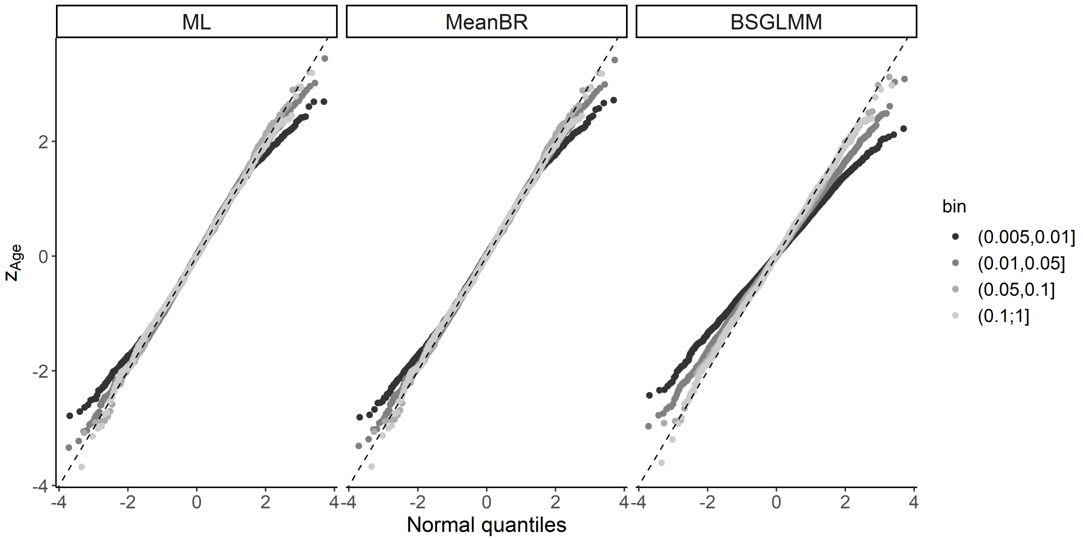

Figure S6: Quantile–quantile (QQ) plots of the quantiles of the simulated data z-scores across bins of voxels versus the theoretical quantiles from a Normal distribution. The lower the lesion incidence (darker colour), the greater the deviations from a linear trend, i.e. the rarer the lesions, the greater the deviations from normality.

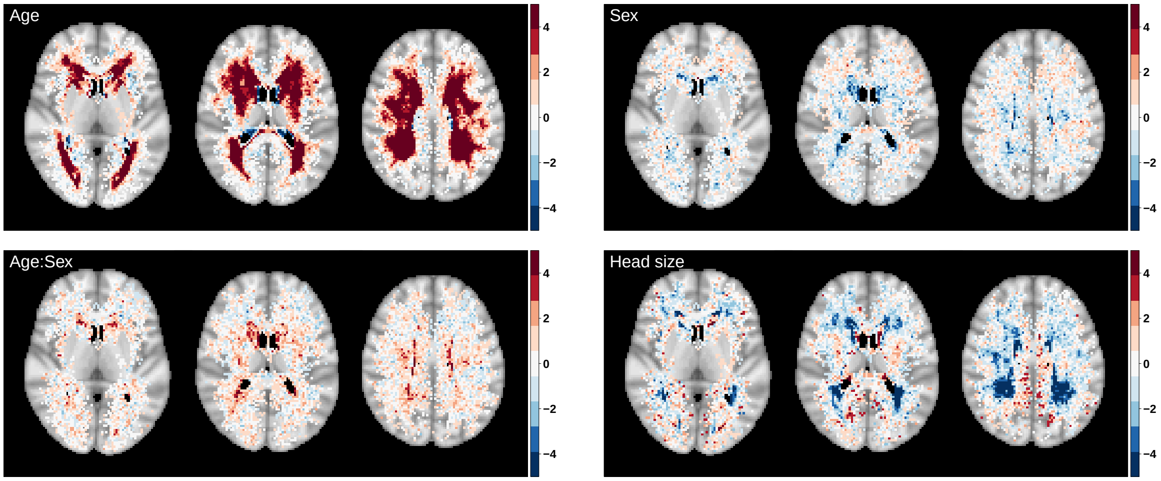

Figure S7: Significance maps (z-scores based on MeanBR estimates) for the effect of age, sex (baseline men), age by sex interaction and head size scaling to complement Figure 5. Data on 13,680 UK Biobank participants used. 72,603 voxels with non-zero lesion probability shown with zero-lesion incidence voxels plotted as transparent to show anatomical MRI for reference; axial slices $\{40, 45, 50\}$ shown.
